# Epigenetics in Abdominal Aortic Aneurysm: Mechanisms and Risk Prediction

**DOI:** 10.64898/2026.01.26.26344463

**Published:** 2026-01-27

**Authors:** Shuai Yuan, Gabrielle Shakt, Michael G. Levin, Katherine Hartmann, Renae Judy, Tia Dinatale, Amy Voorhees, Julie A. Lynch, Saiju Pyarajan, Daniel Levy, Roby Joehanes, VA Million Veteran Program, Kyong-Mi Chang, Philip Tsao, Benjamin F. Voight, Gregory T Jones, Scott M. Damrauer

**Affiliations:** 1 Corporal Michael J. Crescenz VA Medical Center, Philadelphia, PA, USA.; 2 Department of Surgery, University of Pennsylvania Perelman School of Medicine, Philadelphia, PA, USA.; 3 Unit of Cardiovascular and Nutritional Epidemiology, Institute of Environmental Medicine, Karolinska Institutet, Stockholm, Sweden.; 4 Department of Medical Epidemiology and Biostatistics, Karolinska Institutet, Stockholm, Sweden.; 5 Division of Cardiovascular Medicine, Department of Medicine, University of Pennsylvania, Perelman School of Medicine, Philadelphia, PA, USA.; 6 Department of Radiology, Hospital of the University of Pennsylvania, Philadelphia, PA, USA School of Medicine, Philadelphia, PA, USA.; 7 VA Informatics and Computing Infrastructure (VINCI), VA Salt Lake City Heath Care System, Salt Lake City, Utah, USA.; 8 Division of Epidemiology, University of Utah School of Medicine, Salt Lake City, Utah, USA.; 9 Center for Data and Computational Sciences, VA Boston Healthcare System, Boston, MA, USA.; 10 Framingham Heart Study, Framingham, MA, USA; 11 National Heart, Lung, and Blood Institute, National Institutes of Health, Bethesda, MD, USA; 12 Department of Medicine, Perelman School of Medicine, University of Pennsylvania, Philadelphia, PA, USA.; 13 VA Palo Alto Healthcare System, Palo Alto, CA, USA.; 14 Department of Medicine, Stanford University School of Medicine, Stanford, CA, USA.; 15 Stanford Cardiovascular Institute, Stanford University School of Medicine, Stanford, CA, USA.; 16 Department of Genetics, University of Pennsylvania Perelman School of Medicine, Philadelphia, PA, USA.; 17 Department of Systems Pharmacology and Translational Therapeutics, University of Pennsylvania Perelman School of Medicine, Philadelphia, PA, USA.; 18 Institute for Translational Medicine and Therapeutics, University of Pennsylvania Perelman School of Medicine, Philadelphia PA, USA.; 19 Department of Surgical Sciences, University of Otago, Dunedin, New Zealand.

**Keywords:** abdominal aortic aneurysm, DNA methylation, epigenetics, mechanism, prediction

## Abstract

**Background:**

Epigenetic mechanism underlying susceptibility to abdominal aortic aneurysm (AAA) remain poorly understood. Identifying causal DNA methylation markers for AAA can elucidate the regulatory processes that drive aneurysm formation and would accelerate translational applications. We leveraged the VA Million Veteran Program (MVP) to identify methylation biomarkers and delineate underlying pathways.

**Methods:**

We first conducted an epigenome-wide association study (EWAS) of incident AAA (1,324 cases; 42,065 non-cases), performed stratified analyses by population group and smoking status, and conducted Mendelian randomization (MR) to facilitate casual inference of the CpG-AAA association. Chromatin state, island context, and TF binding were implicated through functional annotation of identified CpGs. To identify genes impacted by change in methylation state, we aligned associations with transcriptional data obtained in blood, aorta, and liver. We performed expression quantitative trait methylation (eQTM) to capture CpG–gene-expression links across the genome. Network MR was used to test cardiometabolic mediation. Finally, we developed a risk predictor using methylation data using a penalized regression model, evaluating its performance against a comprehensive clinical model.

**Results:**

EWAS identified 1,253 CpGs associated with incident AAA, and MR supported a putative causal role for 151 of these associations. Functional annotation pointed to predominantly distal, enhancer-centered regulation and enrichment of inflammatory transcription factor programs (e.g., AP-1). This distal architecture was consistent with eQTM results, which showed a larger number of trans associations. Network MR identified 231 putative mediation pathways linking CpGs to AAA, including 179 via cardiometabolic traits and 52 via immune/inflammation-related traits. Among cardiometabolic mediators, blood lipids accounted for >40% of mediation effect linking LDLR-associated CpGs to AAA risk. Among immune/inflammation-related mediators, platelet count and circulating proteins including NEXN, IL1RN, ADH1B, and MMP12 emerged as key intermediates. Genetic colocalization highlighted an aorta-specific cg17511968–WNT6–AAA axis, and network MR implicated IL1RN and MMP12 as downstream protein mediators of association between WNT6-proximal CpGs and AAA. Finally, a methylation risk score improved discrimination when added to a clinical model (AUC 0.775; 95% CI, 0.749–0.801) for incident AAA prediction.

**Conclusions:**

This study identified putative causal DNA methylation markers for AAA, and multi-omics analyses implicate AP-1–linked inflammatory transcriptional programs, blood lipids, platelet count, and multiple immune/inflammation-related proteins as key pathways underlying methylation-associated AAA risk.

## Introduction

Abdominal aortic aneurysm (AAA) is characterized by a focal dilation of the abdominal aorta exceeding 3 cm in diameter. It remains clinically silent until rupture, which carries a mortality rate exceeding 40% upon presentation (1,2). Population screening studies estimate a prevalence of up to 8% in older adults, with incidence rising sharply after age 65 (3-5). Although epidemiological investigations have identified major risk factors—including male sex, advancing age, family history, and particularly cigarette smoking—no pharmacological therapy has been approved to halt aneurysm progression (6); current medical management is limited to controlling cardiovascular risk factors (e.g., antihypertensives, lipid-lowering agents) and surgical repair once the aneurysm reaches a threshold diameter (7).

DNA methylation is a dynamic epigenetic mark that can record cumulative environmental exposures (e.g., smoking, diet, pollution, psychosocial stress) and thereby serves as a molecular integrator of external stimuli over time. These exposure-linked methylation changes can contribute to disease risk by altering gene regulation—modulating transcription factor binding, chromatin accessibility, and promoter/enhancer activity—leading to persistent shifts in gene expression and downstream inflammatory, metabolic, and vascular pathways (8). However, the molecular drivers of AAA pathogenesis, especially epigenetic mechanisms, remain inadequately characterized (9). DNA methylation is a dynamic, environmentally responsive regulator of gene expression that can both reflect disease state and contribute to pathophysiology. An epigenome-wide association study (EWAS) offers an approach to identify differentially methylated regions (i.e. CpGs) associated with incident AAA. By mapping these CpGs to nearby genes and intersecting with gene-regulatory networks, EWAS can uncover novel biomarkers, elucidate pathogenic pathways, and prioritize candidate therapeutic targets (10). Here, we conducted an EWAS integrating genomic data to explore causal CpGs for AAA development and potential pathways and established a methylation score for future AAA risk prediction.

## Methods

### Overview

**Figure 1.**
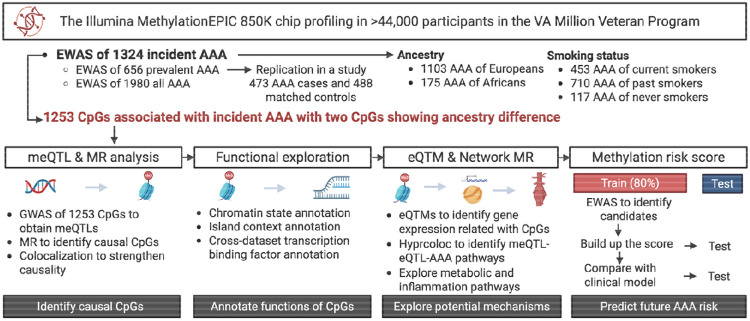
Study design overview. AAA, abdominal aortic aneurysm; eQTM, expression quantitative trait methylation; EWAS, epigenome-wide association study; GWAS, genome-wide association study; meQTL, methylation quantitative trait locus; MR, Mendelian randomization.

Figure 1 outlines the study. We first conducted an EWAS of incident AAA, evaluated population-specific effects, assessed smoking, and contrasted incident vs prevalent associations. To strengthen evidence of causality, we ran genome-wide association study of AAA-associated CpG sites (CpG-GWAS) and used the results in two-sample MR and colocalization experiments. Then, we functionally annotated putative causal CpGs and integrated methylation with gene expression and cardiometabolic and immune/inflammation-related traits to map pathways. Finally, we derived and tested a methylation-based score for predicting incident AAA. The study approved by the VA Central IRB.

### Study population

We performed this study using data from the VA Million Veteran Program (MVP), a large, national cohort of U.S. Veterans established by the Department of Veterans Affairs to elucidate genetic and environmental contributors to human disease (11). This study analyzed MVP participants with available DNA methylation data. Genome-wide methylation was assayed using the Illumina Infinium Methylation EPIC BeadChip array v1 (~850 K probes) (12). Raw intensity data were processed centrally in R using the SeSAMe Bioconductor package for both probe- and sample-level quality control (13). Rigorous quality controls were performed (Supplementary methods).

Age, sex, smoking status (current, past, and never), and AAA diagnoses were ascertained from electronic health records at the time of analysis. Participants with AAA were identified by at least two records of the relevant diagnostic codes (ICD-9: 441.3 or 441.4; ICD-10: I71.3 or I71.4) (14). Comparator participants without AAA lacked these codes and were also excluded if they possessed any related vascular disease codes (ICD-9: 440–448; ICD-10: I71–I75, I77–I79, K55). AAA occurrence was temporally categorized based on the first diagnosis date in the electronic health record, relative to the blood sample collection (enrollment date): prevalent if the diagnosis occurred prior to enrollment, and incident if it occurred after. In the primary analysis, we excluded prevalent AAA cases to minimize the potential for reverse causation. Participants were classified into population groups based on similarity to 1000 Genomes superpopulation reference panels (14). Additional covariates comprised array chip type, scanner ID, storage time (days between blood draw and array hybridization), Houseman-estimated blood cell proportions, and top 10 technical principal components, and top 10 genomic principal components. Diagnosis of hyperlipidemia, hypertension, coronary artery disease, peripheral artery disease, and stroke was defined by self-reported questionnaires and ICDs in electronic health records at enrollment (15).

### EWAS and stratified analyses

Our primary EWAS of incident AAA was performed using logistic regression; covariate specifications are detailed in the Supplementary Methods. To control for statistical inflation, we applied the Bacon method (16); multiple testing was corrected by the Bonferroni procedure, with corrected *P* < 0.05 deemed significant. For CpG–AAA associations identified in this analysis, we evaluated heterogeneity between participants genetically similar to European and African reference populations. Between-population heterogeneity of CpG–AAA associations was assessed using Cochran’s Q statistic and *I^2^*, defining significant heterogeneity as Bonferroni-adjusted Q *P* < 0.05 and *I*^*2*^ > 75%. As internal validation, we tested these CpGs against prevalent AAA and contrasted effect estimates with those for incident AAA. Finally, to assess external validity, we compared our prevalent AAA EWAS results with those from an independent New Zealand case-control study (17).

Given the substantial impact cigarette smoking has on DNA methylation and AAA risk, we analyzed data using using three models to examine its effects with adjustment for 1) self-reported smoking behavior; 2) DNA methylation levels at cg05575921 (*AHRR*); and 3) a methylation score for smoking based on 1255 CpGs (18). We also conducted stratified analyses by smoking status.

### GWAS of DNA methylation biomarkers and MR analysis

To strengthen the evidence for causal associations between CpG methylation and AAA, we conducted two-sample MR, a causal inference method that employs genetic variants as instrumental variables (IVs) (19). To mitigate population structure bias in the downstream MR analysis (since the AAA outcome GWAS primarily comprises data from individuals genetically similar to European reference populations), we performed GWASs for the EWAS-identified CpGs exclusively in individuals genetically similar to the 1000G EUR superpopulation (Supplementary Methods). *Cis* methylation quantitative trait loci (*cis*-meQTL) were selected as IVs at *P* < 5×10^−^ and clumped at *r^2^* < 0.001; F statistics were computed to assess instrument strength and IVs with F < 10 were removed (20). AAA GWAS summary statistics were obtained from a GWAS of 37,214 individuals with AAA (21). Data were harmonized using TwoSampleMR R package (22). CpG-AAA MR associations were estimated using the Wald ratio for single-instrument analyses and the inverse-variance–weighted (IVW) meta-analysis for multiple instruments (fixed-effects model if no heterogeneity; otherwise, random-effects model). MR-PRESSO was applied to detect and correct for horizontal-pleiotropic outliers; outlier-corrected IVW estimates are reported when applicable (23).

### Functional annotations and enrichment analyses

To investigate regulatory functions of AAA-related CpGs, we performed functional annotation across chromatin state, CpG-island context, and transcription factor (TF) binding. We defined three CpG sets: ALL (all EPIC probes used as the array background), EWAS (EWAS-significant CpGs), and MR (MR-significant CpGs). Chromatin states were assigned from Roadmap Epigenomics ChromHMM “mnemonics” tracks for blood, aorta, and liver (24). CpG-island context followed the EPIC manifest. To assess TF involvement, we expanded significant CpGs to ±100 bp windows. TF binding enrichment was evaluated by (i) TF ChIP-seq peak overlap using LOLA (25), and (ii) sequence-motif enrichment using JASPAR 2022 CORE (vertebrates) position-weight matrices with TFBSTools/motifmatchr (26) (Supplementary Methods). We used Fisher’s exact tests to examine differences and controlled the false discovery rate (FDR) Benjamini-Hochberg method for multiple comparisons.

### Genetic colocalization and hyprcoloc analyses

We linked AAA-associated CpGs (±500 kb) to variation associated with cis-gene expression (i.e., expression quantitative trait loci, or eQTLs) using colocalization (27). We then applied HyPrColoc to identify shared meQTL–eQTL–AAA signals (28), where Posterior Probability of Hypothesis 4 is the “shared signal” hypothesis. eQTL data were drawn from whole blood (GTEx v8, eQTLGen), aorta (GTEx v8), and liver (GTEx v8) (29,30). Full model settings are provided in the Supplementary Methods. We defined strong colocalization as PPH3 + PPH4 ≥ 0.7 and PPH4 / (PPH3 + PPH4) ≥ 0.7; HyPrColoc clusters with regional posterior probability of full colocalization (PPFC) ≥ 0.7 were considered colocalized.

### Expression quantitative trait methylation (eQTMs) analysis

An eQTM is a CpG site whose DNA methylation level is associated with the expression level of a nearby (cis) or distant (trans) gene, linking epigenetic variation to transcriptional regulation. Because colocalization targets only *cis* effects, we performed an eQTM analysis to capture both *cis* and *trans* CpG–gene links. Using whole-blood data from 2,115 Framingham Heart Study participants (Supplementary Methods) (31), we tested genome-wide associations between AAA-associated CpGs and genome-wide transcript levels. Multiple testing was controlled by Bonferroni correction.

### Two-stage network MR analysis

We used network MR to test whether cardiometabolic and inflammatory traits mediate CpG–AAA associations (32). First, we estimated effects of genetically predicted CpGs on cardiometabolic and inflammatory traits ($\beta_{\text{CpG}\to \text{mediator}}$) and of genetically predicted cardiometabolic and inflammatory trait on AAA ($\beta_{\text{mediator}\to \text{AAA}}$), as detailed in the Supplementary Methods. Pathways were defined by concordant directionality between total effect ($\beta_{\text{CpG}\to \text{AAA}}$) and indirect effect ($\beta_{\text{CpG}\to \text{mediator}}\times \beta_{\text{mediator}\to \text{AAA}}$). The proportion of the CpG–AAA association mediated by cardiometabolic and inflammatory traits was estimated as $\frac{\beta \text{CpG}\to \text{mediator}\times \beta \text{mediator}\to \text{AAA}}{\beta \text{CpG}\to \text{AAA}}$. Standard errors for these mediated effects were computed via the delta (error-propagation) method (33).

### Methylation score establishment and risk prediction

To derive the AAA methylation score (AAAmeth), we first performed a stratified 80/20 split of incident cases and controls into an independent training (80%) and test (20%) set. All preprocessing was conducted within the training set only to prevent information leakage: CpG beta-values were mean-imputed and standardized, and the resulting imputation and scaling parameters were then applied unchanged to the held-out test set. Within the training set, we fit a LASSO-penalized logistic regression using 10-fold cross-validation to tune the penalty parameter, selecting the 1-SE λ for a parsimonious model. The AAAmeth score was defined as the model’s linear predictor computed from CpGs with non-zero coefficients, and performance was evaluated in the independent 20% test set (34). In the held-out test set, we quantified discrimination (Area Under the Receiver Operating Characteristic Curve [AUC] with 95% CI) and calibration (intercept, slope). We then compared three models: (i) AAAmeth only, (ii) a clinical model including age, sex, ancestry, smoking, BMI, hyperlipidemia, hypertension, and prior diagnoses of coronary artery disease, peripheral artery disease, and stroke, and (iii) the combined model (AAAmeth + clinical covariates). Pairwise AUC differences were tested with the two-sided DeLong test (35).

## Results

### EWAS of incident AAA

**Figure 2.**
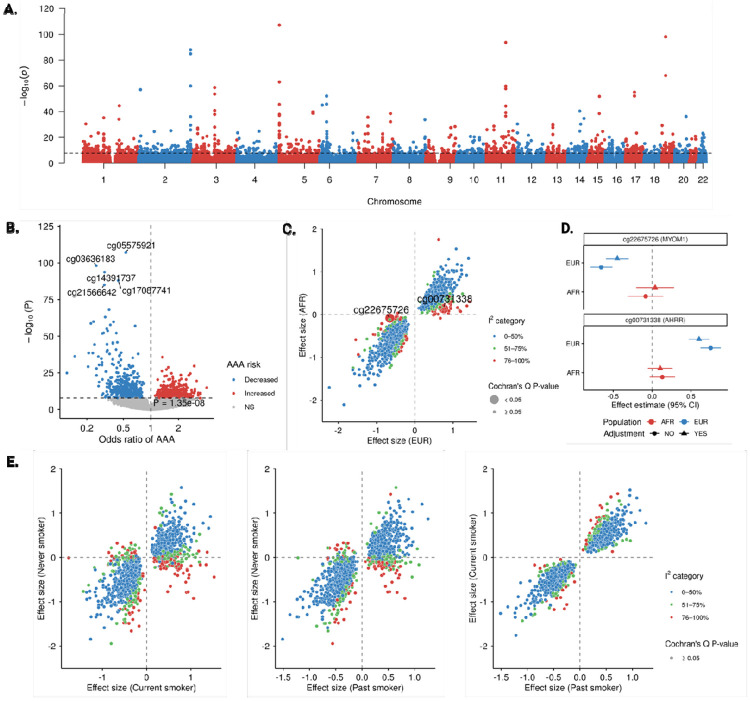
Epigenome-wide association analysis of incident abdominal aortic aneurysm (AAA). A. Manhattan plot of EWAS of incident AAA among 1324 cases and 42,065 controls. The line indicates the significance threshold after Bonferroni correction. B. Volcano plot of CpG-AAA associations. Top five CpGs by p value were labeled. C. Scatter plot of consistency of CpG-AAA associations between individuals of European and African ancestry (determined by genetically similarity to 1000Genomes reference population panels). Two associations (cg22675726-AAA and cg00731338-AAA) were observed with significant heterogeneity after Bonferroni correction. D. Forest plot of cg22675726-AAA and cg00731338-AAA associations with and without smoking status adjustment in individuals of European and African ancestry. E. Scatter plot of consistency of CpG-AAA associations across never, past, and current smokers.

After quality controls, 44,045 of the 48,298 MVP participants with available DNA methylation data were included. Further removing 656 Veterans with prevalent AAA, the EWAS in MVP comprised 1324 Veterans with incident AAA and 42,065 Veterans without incident AAA, testing 757,266 autosomal CpG sites. The analysis included individuals genetically similar to African (n = 11,406), European (n = 26,291), and other superpopulations (n = 6348) reference panels. After Bacon correction, the genomic inflation factor was λ = 1.16, indicating minimal residual inflation. Following Bonferroni adjustment, 1253 CpGs were significantly associated with AAA risk (Figure 2A) after stringent adjustment for age, sex, population structure, and technical PCs; of these, hypermethylation of 545 CpGs and hypomethylation of 708 CpGs were associated with increased risk (Figure 2B; Table S1).

### Population based differences

Overall, we did not observe substantial heterogeneity between analyses in European and African population groups. Among 1253 CpGs, only 2 CpG-AAA associations showed significant heterogeneity between individuals genetically similar to European and African reference populations (Table S2; Figure 2C). Hypomethylation of cg22675726 near the body of the *MYOM1* gene and hypermethylation of cg00731338 near the body of the *AHRR* gene were associated with increased risk of AAA in participants genetically similar to European reference populations but not in those genetically similar to African reference populations (Figure 2D).

### Smoking status difference

Smoking is among the strongest risk factors for AAA and is known to have profound effects on DNA methylation (18). Supporting this, the strongest AAA-associated CpG included cg05575921 (*AHRR*), cg14391737 (*PRSS23*), and cg03636183 (*F2RL3*). Among models with different adjustment, QQ plots showed pronounced inflation in the unadjusted model and the model adjusted for self-reported smoking, whereas models adjusting for *AHRR* methylation or the smoking methylation score lay close to the null (Figure S1). We further evaluated the 1253 CpG–AAA associations separately in never, current, and former smokers. However, we found that the overall pattern of associations appeared similar between current and former smokers (Table S3); heterogeneity tests did not identify significant differences in effect estimates when comparing never vs. current smokers (Table S4) or never vs. former smokers (Table S5) (Figure 2E). We additionally examined several top smoking-related CpGs across the three groups. The effect sizes were directionally consistent in never, current, and former smokers, with wider confidence intervals in never smokers reflecting smaller sample size (Figure S2).

### CpG associations with prevalent vs. incident AAA

We tested whether the 1253 CpGs associated with incident AAA were also associated with prevalent AAA and whether effects differed by case definition. Most effects were concordant; however, 80 CpGs showed significant heterogeneity (Table S6), supporting stage-dependent methylation dynamics rather than a single cross-sectional signal. Notably, hypomethylation was more strongly associated with prevalent AAA in terms of effect size, whereas hypermethylation was more strongly associated with incident AAA.

### Replication of associations for prevalence AAA

We compared the EWAS of prevalent AAA in MVP with results from an external New Zealand male case–control study (473 male cases, 488 matched controls). Of the 316 CpG–AAA associations available in both datasets, 90.8% showed concordant direction of effect (exact binomial sign test vs 0.5: *P* < 2.2×10^−16^, 95% CI 0.88–1.00; Table S7), supporting the robustness and external validity of the MVP findings.

### Mendelian randomization

**Figure 3.**
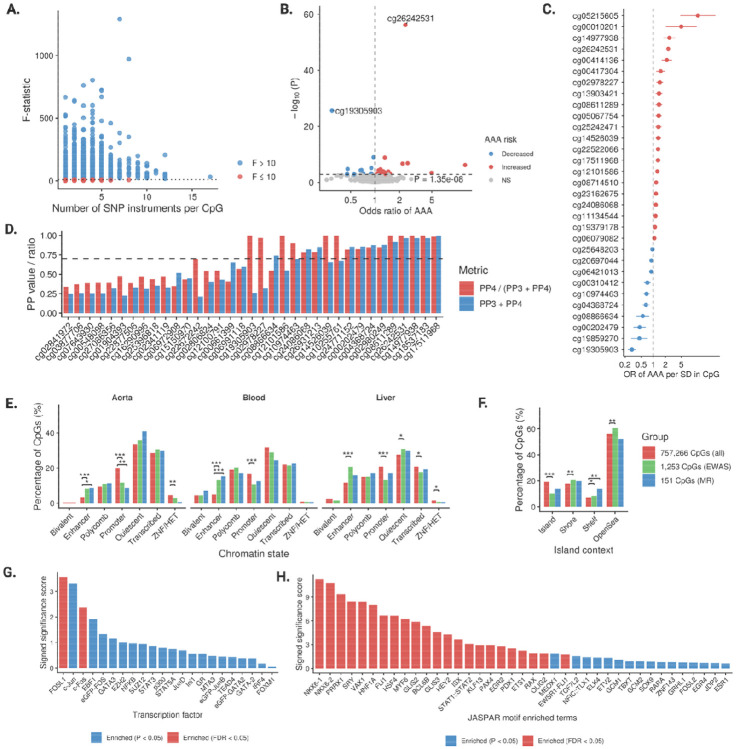
Causal DNA methylation biomarkers associated with AAA and their functional annotations. A. Genetic instruments and their strength in Mendelian randomization analysis. B. Volcano plot of MR associations between genetically predicted levels of CpGs and AAA risk. C. Forest plot of significant CpG-AAA MR associations after FDR correction. D. Colocalization evidence. E. Chromatin state annotation of AAA-associated CpGs against the background CpGs from EpicV2 array. F. Island context annotation of AAA-associated CpGs against the background CpGs from EpicV2 array. G. Roadmap transcription factor binding annotation of causal CpGs. F. JASPAR transcription factor binding annotation of causal CpGs.

GWASs were performed for the 1,253 CpGs associated with incident AAA in up to 25,987 individuals genetically similar to European reference populations. Genetic instruments were available for 1,214 CpGs (*P* < 5×10^−^ ; *r*^*2*^ < 0.001). After excluding 70 CpGs with weak instruments (F < 10), 1,144 CpGs were evaluated in the MR analysis (Figure 3A). Genetically predicted methylation (M-values) at 151 CpGs was associated with AAA at nominal significance (Table S8); far exceeding the 5% expected by chance (exact binomial test one-sided *P* < 2.2×10^−1^ ). After FDR correction, 31 CpGs remained significant (Figure 3B). Effect sizes spanned from an OR of 0.29 (95% CI, 0.24–0.37) for hypermethylation at cg19305903 (*AHRR*, body) to an OR of 12.8 (95% CI, 4.70–34.95) for hypermethylation at cg05215605 (*CDK6*, body) (Figure 3C). Eleven CpGs showed strong colocalization with AAA, supporting shared causal variants (Figure 2D). Among the 151 CpGs, several lay within or near established AAA risk loci (21) (lead variant position ± 250kb) encompassing previously prioritized AAA genes, including lipid-related genes (*HMGCR, LDLR, TRIB1, EPHX2*), immune/inflammation-related gens (*CDK6, ERG, PLAUR, MFAP2, ZBTB46, SPSB1, TGFBR3*), and a non-clear cluster (*ADH1C, LHFPL2, ODF3, SUGCT, ZNF827*) (Figure S3).

### Functional annotation and pathway enrichment

Across aorta, blood, and liver, CpGs associated with AAA (by EWAS or MR) were consistently enriched in enhancer chromatin and depleted at promoters relative to the EPIC array background (Figure 3E). By island context, both EWAS and MR sets were depleted in CpG islands and enriched in shelves (Figure 3F). For TF binding, LOLA ChIP–seq overlaps highlighted AP-1 (FOS/JUN) as the top signal, remaining significant after FDR correction (Figure 3G). Independent motif scanning reproduced this pattern, showing strong enrichment of AP-1 and additional immune/inflammatory modules (STAT/IRF, ETS/KLF/GATA; Figure 3H).

### Gene expression pathways linking CpG-AAA

**Figure 4.**
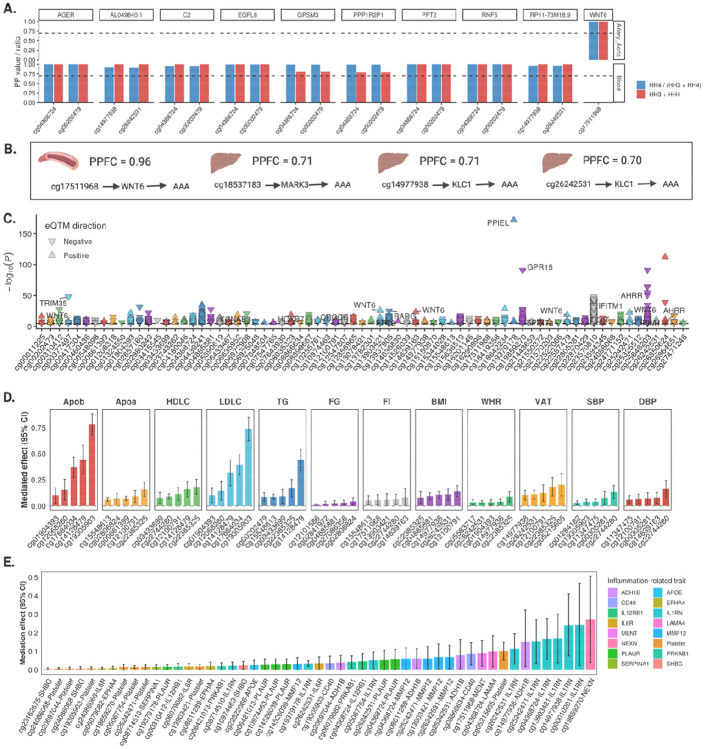
Gene expression and cardiometabolic pathways supporting CpG-AAA links. A. Gene expression associated with AAA-associated CpGs based on eQTM dataset. B. Gene expression colocalized with meQTLs at the cis region in blood, artery aorta, or liver tissues. C. meQTL-eQTL-AAA associations prioritized by Hyprcoloc analysis in blood, artery aorta, or liver tissues. D. Cardiometabolic pathways linking CpGs to AAA and their mediation magnitude. E. Immune/inflammation-related pathways linking CpGs to AAA and their mediation magnitude.

A total of 259 CpG-gene pairs (including 78 unique CpGs and 180 unique genes) had strong colocalization support across blood, aorta, and liver (Table S10). Among the 11 CpGs that had both significant MR results and colocalization with AAA, 10 genes also colocalized with gene expression in blood or aorta—including *AGER, C2, WNT6,* and *RNF5* (Figure 4A). HyPrColoc identified four multi-trait clusters with PPFC ≥ 0.7 (Figure 4B); notably, the cg17511968–*WNT6*–AAA pathway was supported by multiple lines of evidence.

### Genome-wide links between AAA CpGs and gene expression

eQTM analyses were performed to estimate the correlations between the AAA-associated CpGs and gene expression across the genome in 2,115 Framingham Heart Study participants. These analyses revealed that while only small proportion of the AAA-associated CpGs (0.7%) map to transcripts in *cis* (within the same genomic region), the majority demonstrated a linkage to a larger number of genes in *trans* (at distant genomic locations) (Figure 4C; Table S11). Several trans-linked genes (*CDKN1A, DAB2IP, EPHX2, IFIH1, LOXL1, MAP3K20, NFKB1, NICN1, SCARB1, SH3RF3, SMAD3, SPSB1, ZNF827*) have been priorly identified in a large-scale AAA GWAS (21) (Table S11).

### Cardiometabolic and immune signaling pathways linking CpGs to AAA

Because AAA is driven by atherosclerotic burden and immune-mediated vascular remodeling, we performed two-stage network MR to evaluate whether cardiometabolic traits and immune/inflammatory signaling proteins mediate CpG–AAA associations. After FDR correction, 12 genetically proxied cardiometabolic traits, genetically proxied platelet count, and 25 genetically proxied inflammatory blood proteins were associated with genetic liability AAA and were considered in the network MR analyses (Table S12). Among 151 AAA-associated CpGs in MR, genetically predicted methylation of 105 CpGs was associated with at least one of 12 genetically proxied cardiometabolic traits, yielding 305 CpG–cardiometabolic trait pairs (Table S13). For inflammatory traits, genetically predicted methylation of 51 CpGs was associated with genetically proxied platelet count and genetically predicted methylation of 145 CpGs was associated with at least one of 52 genetically proxied immune/inflammation-related proteins, yielding 1016 CpG–inflammation-related trait pairs (Table S14). We identified 231 putative mediation pathways based on concordant directionality between the total effect and indirect effect. Of these, 179 involved cardiometabolic mediators (Table S15) and 52 involved immune/inflammation-related mediators (Table S16). Among cardiometabolic traits, blood lipids including apolipoprotein B, low-density lipoprotein cholesterol, and triglycerides mediated substantial proportions (>40%) of the association for several CpGs (i.e., cg19305903, cg17684034, cg14128479; Figure 4D). Visceral adipose tissue percentage and BMI also mediated CpGs in *CDK6* and *ZFYVE21*, but with smaller effects (Figure 4D). For inflammation-related pathways, IL1RN, ADH1B, MMP12 proteins mediated several CpG-AAA associations. (Figure 4E). NEXN protein mediated 27.3% (95% CI 4.1%-50.5%) of cg19859270-AAA association and platelet count mediated 10.1 (95% CI, 5.3%-14.9%) of cg05215605-AAA association (Figure 4E).

### AAA methylation score for future AAA risk prediction

**Figure 5.**
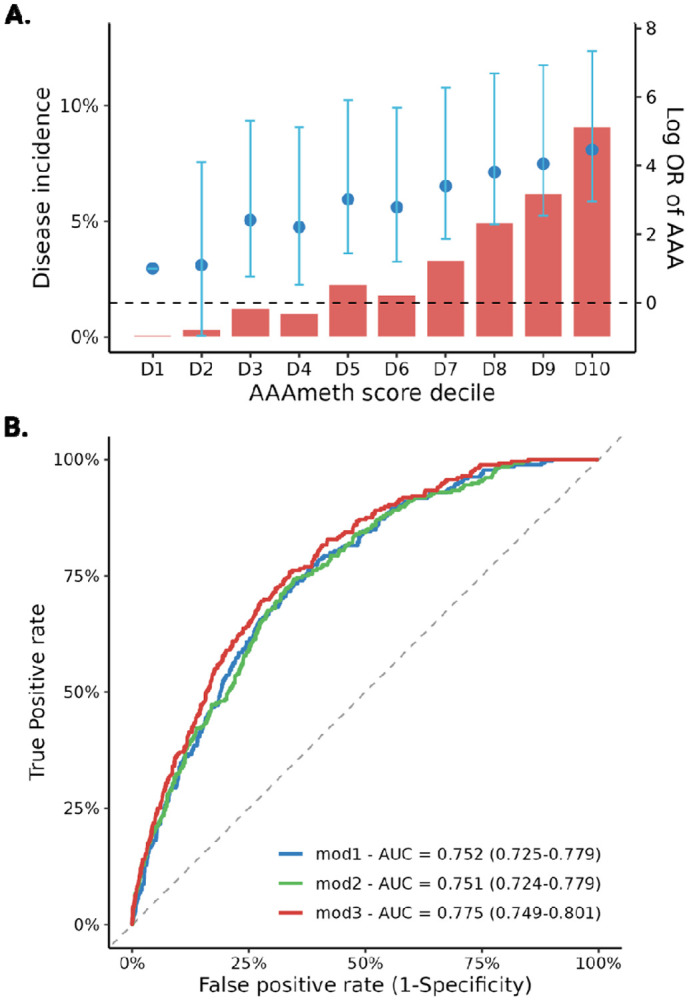
The AAA methylation score in relation to incident AAA and its prediction ability. A. Incidence and odds of developing AAA by decile of the AAA methylation score. B. Area Under the Receiver Operating Characteristic Curve (AUC) of different prediction models (mod 1 – AAAmeth score; mod 2 – clinical score, including age in years, sex[men or women] , ancestry [European, African, or other], smoking status [never, past, or current smoker], body mass index in kg/m^2^, diagnosis of hyperlipidemia, hypertension, coronary artery disease, peripheral artery disease, or stroke[yes or no]).

We randomly assigned 1,059 individuals with incident AAA and 33,652 individuals without incident AAA to the training set and 265 individuals with incident and 8,413 individuals without incident AAA to the test set. Using LASSO-penalized logistic regression with 10-fold, 10-repeat cross-validation and a 1-SE penalty (λ_1_SE = 0.002), the model retained 87 CpGs for the AAA methylation score (training AUC = 0.791). The score was computed as the linear predictor from these coefficients (weights in Table S17) and was positively associated with incident AAA: OR per SD = 1.53 (95% CI, 1.42–1.63; Figure 5A). In the test set, discrimination was AUC = 0.752 (95% CI, 0.725–0.779), comparable to a comprehensive clinical model (AUC = 0.751; 95% CI, 0.724–0.779; DeLong *P* = 0.93). Combining the methylation and clinical covariates improved performance (AUC = 0.775; 95% CI, 0.749–0.801), outperforming both the AAA methylation score alone (DeLong *P* < 0.001) and the clinical model (DeLong *P* = 0.005; Figure 5B). The combined model had a calibration intercept of 0.021 and a slope of 0.873, with Brier = 0.029; after probability calibration, the intercept was 0.021 and the slope was 0.89, with Brier = 0.029.

## Discussion

This large-scale EWAS identified 1,253 CpGs associated with AAA in observational analyses and obtained genetic support for causality for more than 100 CpG–AAA associations. Population-stratified analyses showed minimal heterogeneity in CpG–AAA associations between individuals genetically similar to European and African reference populations. Functional annotation implicated predominantly distal, enhancer-centered regulation and highlighted key inflammatory transcription factor programs. Mediation analyses indicated that several key gene expression, blood lipids, and several inflammation-related traits (proteins NEXN, IL1RN, ADH1B, MMP12, and platelet count) partially explain the CpG–AAA relationships. A methylation-based AAA risk score improved prediction of future AAA beyond a clinical model, indicating added, non-redundant information. Collectively, these findings elucidate epigenetic mechanisms underlying AAA and provide deeper insight into disease etiology and opportunities for prevention and treatment.

Smoking, a major risk factor for AAA, is also a dominant driver of DNA methylation (18). Adjustment for AHRR methylation or a smoking methylation score markedly attenuated CpG–AAA associations, indicating that much of the signal lies on a smoking-related methylation axis and is consistent with partial mediation. In contrast, stratified analyses by self-reported smoking status showed no strong interaction, with broadly similar CpG–AAA effect estimates across never, former, and current smokers. Together, these findings suggest that smoking-related methylation underpins a substantial proportion of the CpG–AAA signal, while the relative CpG–AAA association is similar across smoking categories and not fully captured by crude self-report. Accordingly, CpG profiles generally appear to record a durable smoking-related epigenetic imprint that contributes to AAA risk and may serve as an integrated biomarker of vascular risk.

Our pathway analyses implicated lipid metabolism and adiposity as key cardiometabolic axes linking CpG methylation to AAA. This conclusion was supported by two independent lines of evidence. First, several lipid-related genes (*LDLR, HMGCR, TRIB1*) and the adiposity-related gene *CDK6* mapped in cis to AAA-associated CpGs. Second, MR-based mediation analyses showed that methylation at cg19305903, cg17684034, and cg14128479 in these loci was associated with AAA, with much of the effect mediated through apolipoprotein B, low-density lipoprotein cholesterol, and triglycerides, and that the cg05215605 (*CDK6*)–AAA association was mediated by visceral adipose tissue (36). Together, these findings strengthen the case for a causal contribution of selected CpGs, prioritize druggable lipid and adiposity pathways for mechanistic follow-up. In turn, they underscore lipids and central adiposity as primary cardiometabolic drivers of AAA, with direct implications for risk stratification and for targeting lipid- and adiposity-lowering interventions in AAA prevention.

Integrating functional annotation with eQTM, we find that multiple AAA-associated CpGs preferentially localize to distal regulatory elements and align with AP-1–centered immune/inflammatory transcriptional programs (37). AP-1 (FOS/JUN family) is a canonical stress- and cytokine-responsive transcription factor complex downstream of MAPK signaling (JNK, p38, ERK) that regulates a broad set of genes involved in leukocyte activation, cytokine production, and matrix remodeling, all processes that have been shown to be central to aneurysm formation in model systems (38,39). Consistent with distal control, eQTM analyses show a predominance of trans CpG–gene links. Together these data suggest that methylation changes at enhancer-like CpGs influence AP-1 and cooperating TF hubs (e.g., STAT/IRF) amplify downstream transcriptional responses across multiple loci. This architecture nominates several potential therapeutic targets, including upstream MAPK kinases (e.g., JNK/p38 inhibitors), modulators of inflammatory cytokine signaling that converge on AP-1 (e.g., IL-6 pathway inhibitors), and epigenetic regulators of enhancer activity (e.g., BET or HDAC inhibitors) as candidates to dampen AP-1–driven programs in AAA (40,41). We note, however, that features such as 3D chromatin contacts, cell-state composition, and shared upstream drivers could also contribute to the observed trans architecture and should be interrogated in future mechanistic studies.

In the immune/inflammation arm of the network MR, we highlighted several plausible mediators, including platelet count and circulating proteins related to vascular remodeling and inflammatory signaling (NEXN, IL1RN, ADH1B, and MMP12). These pathways strengthen the biological plausibility of the CpG–AAA associations by adding mechanistic support, rather than relying on statistical association alone. For example, our prior work implicated MMP12 as a key mediator linking smoking to AAA (42). Consistent with that, the current analysis suggests that several smoking-associated CpGs (cg26242531, cg13903421, and cg25242471) (43) may influence AAA risk through effects on MMP12 levels. Together, these findings support a coherent model in which smoking alters DNA methylation at specific loci, which in turn perturb MMP12 and contributes to aneurysm risk. Beyond mechanism, these results can help prioritize therapeutic hypotheses. Notably, IL1RN emerged as a recurrent immune-related mediator linking CpGs to AAA, pointing to the IL-1 pathway as a potential intervention axis. This does not prove that IL-1 blockade will prevent AAA, but it provides a rationale to evaluate IL-1–targeted therapies (e.g., anakinra) in high-risk individuals or in appropriately designed clinical studies, with careful attention to patient selection and safety (44).

Tissue-level gene expression data were interrogated to explore mechanisms. Although >100 genes showed *cis* colocalization with AAA-related CpGs in relevant tissues, relatively few overlapped established AAA GWAS signals (21) likely reflecting tissue specificity, limited power or imperfect gene prioritization at loci identified in GWAS, or regulation driven by both genetic and environmental inputs. Notably, two AAA-colocalized CpGs (cg02980249 and cg04368724) colocalized with AAA-related genes within the *MHC* class III region (*AGER* [also known as *RAGE*], *C2*, and *RNF5*) which also have experimental or human-tissue support in AAA and are linked to inflammatory pathways (45-47). These findings together suggest that methylation at cg02980249 and cg04368724 may modulate inflammatory responses that, in turn, contribute to AAA development.

One notable pathway not highlighted by prior AAA GWAS is the aorta-specific cg17511968–WNT6–AAA axis. Although WNT6 itself has not emerged as a lead GWAS locus, the broader Wnt/β-catenin signaling cascade is repeatedly implicated in aneurysm biology, with both human and murine aneurysmal aortas showing increased pathway activation compared with controls (48). Two experimental studies further support this connection. First, low-dose colchicine inhibited aneurysm formation in human and mouse models by increasing sclerostin (SOST), thereby suppressing Wnt/β-catenin signaling in vascular smooth muscle cells (49); Second, SOST was downregulated in human and murine AAA tissue, and restoring SOST (via transgenic expression or recombinant protein) attenuated AngII-induced aneurysm formation and inflammatory readouts, again consistent with pathogenic activation of Wnt/β-catenin signaling in AAA (50). Beyond vascular smooth muscle biology, WNT6 has also been implicated in macrophage polarization (51), a process with well-established relevance to AAA development and progression (52). In our network MR analyses, we additionally observed evidence that CpGs in the WNT6 vicinity may influence AAA risk through immune-related mediators, including IL1RN and MMP12. Taken together, these lines of evidence point to WNT6-associated regulatory pathways—potentially operating through Wnt/β-catenin activity and downstream inflammatory remodeling—as an underappreciated component of AAA pathogenesis.

Prior studies in a mouse AAA model and cultured human smooth muscle cells have implicated promoter methylation of genes such as *IL6R, MMP9,* and *SMYD2* in AAA (53)(54). In our data, methylation at CpG sites located in the promoter or gene body of these loci was associated with incident AAA at nominal levels of significance, although none surpassed the epigenome-wide multiple testing threshold. A likely explanation is tissue context: earlier studies profiled vascular smooth muscle cells, whereas our analyses were conducted in whole blood. Consistent with this, a previous blood-based methylation study likewise did not observe strong *MMP9* signals (55).

We developed the first methylation-based risk score for incident AAA (AAAmeth) and evaluated its predictive performance. In the test set, our methylation score achieved AUC 0.752 and outperformed the best polygenic risk score (AUC 0.708) (56). Discrimination was similar to a comprehensive clinical model (AUC 0.751). Notably, combining AAAmeth with the clinical covariates improved AUC to 0.775, significantly better than methylation alone and the clinical model, indicating that methylation score contributes non-redundant information for AAA risk prediction. However, whether the score is portable across settings remains to be determined.

Several caveats warrant consideration. First, our EWAS relies on whole-blood methylation, which constrains tissue-level interpretation despite complementary tissue-specific gene-related analyses. Second, the VA MVP cohort is predominantly male veterans with unique exposures and health profiles; residual confounding may affect internal validity and selection mechanisms may limit external generalizability. We mitigated bias by prespecifying covariates, controlling test-statistic inflation, and applying MR to reduce confounding. We also observed concordance with an external case-control study; nonetheless, generalizability remains uncertain. Third, MR was conducted in individuals genetically similar to European reference populations due to data availability, although stratified EWAS suggested broadly similar CpG–AAA associations in those similar to African reference populations; ancestry-diverse MR will be needed. Fourth, even with large data sources, limited power could yield false negatives. Fifth, the eQTM analyses utilized the older Infinium HumanMethylation450 (450k) array (31), which resulted in coverage that did not include all CpGs identified in our analysis; consequently, eQTM data were unavailable for a subset of our CpGs. Sixth, overfitting of the methylation score remains possible and warrants validation in independent datasets. Finally, all evidence here is generated from in-silico analyses; orthogonal validation in experimental models and prospective clinical studies is required to establish mechanism and translational utility.

In summary, this large-scale EWAS, integrated with genetic and multi-omic analyses, identifies blood DNA methylation markers for incident AAA with supportive causal evidence. Mechanistic analyses implicate distal, enhancer-centric, inflammation-linked regulation and point to several gene-expression, lipid and inflammation-related pathways as mediators. We also introduce a methylation-based risk score that complements clinical factors, though its external applicability and clinical utility require prospective validation. Collectively, these findings establish an epigenetic framework for refining AAA risk prediction and prioritizing molecular targets for prevention and therapy.

## Supplementary Material

Supplement 1

Supplement 2

## Data Availability

The MVP individual-level data are open to VA-affiliated researchers via application (https://www.mvp.va.gov/pwa/mvp-data-available-research). The generated EWAS results and summary GWAS data on CpG probes will be uploaded to dbGAP once when the paper has been published.

## References

[1] Hoornweg LL, Storm-Versloot MN, Ubbink DT, Koelemay MJW, Legemate DA, Balm R. Meta analysis on mortality of ruptured abdominal aortic aneurysms. Eur J Vasc Endovasc Surg. 2008 May;35(5):558–70. 18226567 10.1016/j.ejvs.2007.11.019

[2] Abdulameer H, Al Taii H, Al-Kindi SG, Milner R. Epidemiology of fatal ruptured aortic aneurysms in the United States (1999-2016). J Vasc Surg. 2019 Feb;69(2):378–384.e2. 29960790 10.1016/j.jvs.2018.03.435

[3] Marcaccio CL, Schermerhorn ML. Epidemiology of abdominal aortic aneurysms. Semin Vasc Surg. 2021 Mar;34(1):29–37. 33757632 10.1053/j.semvascsurg.2021.02.004

[4] Grøndal N, Søgaard R, Lindholt JS. Baseline prevalence of abdominal aortic aneurysm, peripheral arterial disease and hypertension in men aged 65-74 years from a population screening study (VIVA trial). Br J Surg. 2015 July;102(8):902–6. 25923784 10.1002/bjs.9825

[5] Ashton HA, Buxton MJ, Day NE, Kim LG, Marteau TM, Scott R a. P, The Multicentre Aneurysm Screening Study (MASS) into the effect of abdominal aortic aneurysm screening on mortality in men: a randomised controlled trial. Lancet. 2002 Nov 16;360(9345):1531–9. 12443589 10.1016/s0140-6736(02)11522-4

[6] Sakalihasan N, Michel JB, Katsargyris A, Kuivaniemi H, Defraigne JO, Nchimi A, Abdominal aortic aneurysms. Nat Rev Dis Primers. 2018 Oct 18;4(1):34. 30337540 10.1038/s41572-018-0030-7

[7] Golledge J. Abdominal aortic aneurysm: update on pathogenesis and medical treatments. Nat Rev Cardiol. 2019 Apr;16(4):225–42. 30443031 10.1038/s41569-018-0114-9

[8] Smith ZD, Hetzel S, Meissner A. DNA methylation in mammalian development and disease. Nat Rev Genet. 2025 Jan;26(1):7–30. 39134824 10.1038/s41576-024-00760-8

[9] Gurung R, Choong AM, Woo CC, Foo R, Sorokin V. Genetic and Epigenetic Mechanisms Underlying Vascular Smooth Muscle Cell Phenotypic Modulation in Abdominal Aortic Aneurysm. Int J Mol Sci. 2020 Aug 31;21(17):6334. 32878347 10.3390/ijms21176334 PMC7504666

[10] Wei S, Tao J, Xu J, Chen X, Wang Z, Zhang N, Ten Years of EWAS. Adv Sci (Weinh). 2021 Oct;8(20):e2100727. 34382344 10.1002/advs.202100727 PMC8529436

[11] Verma A, Huffman JE, Rodriguez A, Conery M, Liu M, Ho YL, Diversity and scale: Genetic architecture of 2068 traits in the VA Million Veteran Program. Science. 2024 July 19;385(6706):eadj1182. 39024449 10.1126/science.adj1182 PMC12857194

[12] Noguera-Castells A, García-Prieto CA, Álvarez-Errico D, Esteller M. Validation of the new EPIC DNA methylation microarray (900K EPIC v2) for high-throughput profiling of the human DNA methylome. Epigenetics. 2023 Dec 31;18(1):2185742. 36871255 10.1080/15592294.2023.2185742 PMC9988339

[13] Zhou W, Triche TJ, Laird PW, Shen H. SeSAMe: reducing artifactual detection of DNA methylation by Infinium BeadChips in genomic deletions. Nucleic Acids Res. 2018 Nov 16;46(20):e123. 30085201 10.1093/nar/gky691 PMC6237738

[14] 1000 Genomes Project Consortium, Auton A, Brooks LD, Durbin RM, Garrison EP, Kang HM, A global reference for human genetic variation. Nature. 2015 Oct 1;526(7571):68–74. 26432245 10.1038/nature15393 PMC4750478

[15] Klarin D, Tsao PS, Damrauer SM. Genetic Determinants of Peripheral Artery Disease. Circulation Research. 2021 June 11;128(12):1805–17. 34110906 10.1161/CIRCRESAHA.121.318327

[16] van Iterson M, van Zwet EW, Heijmans BT, the BIOS Consortium. Controlling bias and inflation in epigenome- and transcriptome-wide association studies using the empirical null distribution. Genome Biology. 2017 Jan 27;18(1):19. 28129774 10.1186/s13059-016-1131-9 PMC5273857

[17] Jones GT, Williams MJA, Khashram M, Fitzgerald S, Lyons OTA, Griffin CL, A DNA Methylation Marker, cg05575921 (AHRR), Outperforms Self-Reported Smoking Exposure for Its Association With Cardiovascular Disease Prevalence. Nicotine Tob Res. 2025 Aug 5;ntaf165. 10.1093/ntr/ntaf165 40849734

[18] Chybowska AD, Bernabeu E, Yousefi P, Suderman M, Hillary RF, Clark R, A blood- and brain-based EWAS of smoking. Nat Commun. 2025 Apr 4;16(1):3210. 40180905 10.1038/s41467-025-58357-6 PMC11968855

[19] Sanderson E, Glymour MM, Holmes MV, Kang H, Morrison J, Munafò MR, Mendelian randomization. Nat Rev Methods Primers. 2022 Feb 10;2:6. 37325194 10.1038/s43586-021-00092-5 PMC7614635

[20] Burgess S, Davies NM, Thompson SG. Bias due to participant overlap in two-sample Mendelian randomization. Genet Epidemiol. 2016 Nov;40(7):597–608. 27625185 10.1002/gepi.21998 PMC5082560

[21] Roychowdhury T, Klarin D, Levin MG, Spin JM, Rhee YH, Deng A, Genome-wide association meta-analysis identifies risk loci for abdominal aortic aneurysm and highlights PCSK9 as a therapeutic target. Nat Genet. 2023 Nov;55(11):1831–42. 37845353 10.1038/s41588-023-01510-y PMC10632148

[22] Hemani G, Zheng J, Elsworth B, Wade KH, Haberland V, Baird D, The MR-Base platform supports systematic causal inference across the human phenome. Elife. 2018 May 30;7:e34408. 29846171 10.7554/eLife.34408 PMC5976434

[23] Verbanck M, Chen CY, Neale B, Do R. Detection of widespread horizontal pleiotropy in causal relationships inferred from Mendelian randomization between complex traits and diseases. Nat Genet. 2018 May;50(5):693–8. 29686387 10.1038/s41588-018-0099-7 PMC6083837

[24] Herzig S, Long F, Jhala US, Hedrick S, Quinn R, Bauer A, CREB regulates hepatic gluconeogenesis through the coactivator PGC-1. Nature. 2001 Sept 13;413(6852):179–83. 11557984 10.1038/35093131

[25] Sheffield NC, Bock C. LOLA: enrichment analysis for genomic region sets and regulatory elements in R and Bioconductor. Bioinformatics. 2016 Feb 15;32(4):587–9. 26508757 10.1093/bioinformatics/btv612 PMC4743627

[26] Tan G, Lenhard B. TFBSTools: an R/bioconductor package for transcription factor binding site analysis. Bioinformatics. 2016 May 15;32(10):1555–6. 26794315 10.1093/bioinformatics/btw024 PMC4866524

[27] Giambartolomei C, Vukcevic D, Schadt EE, Franke L, Hingorani AD, Wallace C, Bayesian test for colocalisation between pairs of genetic association studies using summary statistics. PLoS Genet. 2014 May;10(5):e1004383. 24830394 10.1371/journal.pgen.1004383 PMC4022491

[28] Foley CN, Staley JR, Breen PG, Sun BB, Kirk PDW, Burgess S, A fast and efficient colocalization algorithm for identifying shared genetic risk factors across multiple traits. Nat Commun. 2021 Feb 3;12(1):764. 33536417 10.1038/s41467-020-20885-8 PMC7858636

[29] Võsa U, Claringbould A, Westra HJ, Bonder MJ, Deelen P, Zeng B, Large-scale cis- and trans-eQTL analyses identify thousands of genetic loci and polygenic scores that regulate blood gene expression. Nat Genet. 2021 Sept;53(9):1300–10. 34475573 10.1038/s41588-021-00913-z PMC8432599

[30] GTEx Consortium. The GTEx Consortium atlas of genetic regulatory effects across human tissues. Science. 2020 Sept 11;369(6509):1318–30. 32913098 10.1126/science.aaz1776 PMC7737656

[31] Keshawarz A, Bui H, Joehanes R, Ma J, Liu C, Huan T, Expression quantitative trait methylation analysis elucidates gene regulatory effects of DNA methylation: the Framingham Heart Study. Sci Rep. 2023 Aug 10;13(1):12952. 37563237 10.1038/s41598-023-39936-3 PMC10415314

[32] Burgess S, Daniel RM, Butterworth AS, Thompson SG, EPIC-InterAct Consortium. Network Mendelian randomization: using genetic variants as instrumental variables to investigate mediation in causal pathways. Int J Epidemiol. 2015 Apr;44(2):484–95. 25150977 10.1093/ije/dyu176 PMC4469795

[33] Larsson SC, Woolf B, Gill D. Appraisal of the causal effect of plasma caffeine on adiposity, type 2 diabetes, and cardiovascular disease: two sample mendelian randomisation study. bmjmed [Internet]. 2023 Mar 14 [cited 2025 May 31];2(1). Available from: https://bmjmedicine.bmj.com/content/2/1/e000335 10.1136/bmjmed-2022-000335 PMC9978685 36936261

[34] Ranstam J, Cook JA. LASSO regression. Br J Surg. 2018 Sept 1;105(10):1348.

[35] DeLong ER, DeLong DM, Clarke-Pearson DL. Comparing the areas under two or more correlated receiver operating characteristic curves: a nonparametric approach. Biometrics. 1988 Sept;44(3):837–45. 3203132

[36] Hou X, Zhang Y, Li W, Hu AJ, Luo C, Zhou W, CDK6 inhibits white to beige fat transition by suppressing RUNX1. Nat Commun. 2018 Mar 9;9(1):1023. 29523786 10.1038/s41467-018-03451-1 PMC5845007

[37] Li H, Bai S, Ao Q, Wang X, Tian X, Li X, Modulation of Immune-Inflammatory Responses in Abdominal Aortic Aneurysm: Emerging Molecular Targets. J Immunol Res. 2018 June 3;2018:7213760. 29967801 10.1155/2018/7213760 PMC6008668

[38] Li Z, Kong W. Cellular signaling in Abdominal Aortic Aneurysm. Cellular Signalling. 2020 June 1;70:109575. 32088371 10.1016/j.cellsig.2020.109575

[39] Sun D, Zhang M, Li Y, Mei S, Qin J, Yan J. c-Jun/Ap-1 is upregulated in an Ang II-induced abdominal aortic aneurysm formation model and mediates Chop expression in mouse aortic smooth muscle cells. Mol Med Rep. 2019 May;19(5):3459–68. 30864718 10.3892/mmr.2019.10017 PMC6472129

[40] Yan H, Cui B, Zhang X, Fu X, Yan J, Wang X, Antagonism of toll-like receptor 2 attenuates the formation and progression of abdominal aortic aneurysm. Acta Pharm Sin B. 2015 May;5(3):176–87. 26579444 10.1016/j.apsb.2015.03.007 PMC4629243

[41] Lindeman JHN, Abdul-Hussien H, van Bockel JH, Wolterbeek R, Kleemann R. Clinical trial of doxycycline for matrix metalloproteinase-9 inhibition in patients with an abdominal aneurysm: doxycycline selectively depletes aortic wall neutrophils and cytotoxic T cells. Circulation. 2009 Apr 28;119(16):2209–16. 19364980 10.1161/CIRCULATIONAHA.108.806505

[42] Yuan S, Khodursky S, Geng J, Sharma P, Spin JM, Tsao PS, Circulating Protein Mediators Linking Genetically Predicted Smoking to Abdominal Aortic Aneurysm: A Genomic-Proteomic Analysis. Arteriosclerosis, Thrombosis, and Vascular Biology. 2025 Sept;45(9):1683–92. 40671653 10.1161/ATVBAHA.125.323057 PMC12285744

[43] Joehanes R, Just AC, Marioni RE, Pilling LC, Reynolds LM, Mandaviya PR, Epigenetic Signatures of Cigarette Smoking. Circ Cardiovasc Genet. 2016 Oct;9(5):436–47. 27651444 10.1161/CIRCGENETICS.116.001506 PMC5267325

[44] Johnston WF, Salmon M, Pope NH, Meher A, Su G, Stone ML, Inhibition of Interleukin-1β Decreases Aneurysm Formation and Progression in a Novel Model of Thoracic Aortic Aneurysms. Circulation. 2014 Sept 9;130(11 Suppl 1):S51–9. 25200056 10.1161/CIRCULATIONAHA.113.006800 PMC5097450

[45] Hinterseher I, Erdman R, Donoso LA, Vrabec TR, Schworer CM, Lillvis JH, Role of Complement Cascade in Abdominal Aortic Aneurysms. Arteriosclerosis, Thrombosis, and Vascular Biology. 2011 July;31(7):1653–60. 21493888 10.1161/ATVBAHA.111.227652 PMC3712630

[46] Zhang F, Kent KC, Yamanouchi D, Zhang Y, Kato K, Tsai S, Anti-receptor for advanced glycation end products therapies as novel treatment for abdominal aortic aneurysm. Ann Surg. 2009 Sept;250(3):416–23. 19652591 10.1097/SLA.0b013e3181b41a18 PMC2921961

[47] Bi C, Liu B, Gao P, Wang C, Fang S, Huo Z, RAGE deficiency ameliorates abdominal aortic aneurysm progression. Inflamm Res. 2025 Apr 17;74(1):63. 40244438 10.1007/s00011-025-02027-2

[48] Puertas-Umbert L, Varona S, Ballester-Servera C, Alonso J, Aguiló S, Orriols M, Activation of Wnt/β-catenin signaling in abdominal aortic aneurysm: A potential therapeutic opportunity? Genes & Diseases. 2023 May 1;10(3):639–42. 37396504 10.1016/j.gendis.2022.05.017 PMC10308103

[49] Chen M, Yang D, Zhou Y, Yang C, Lin W, Li J, Colchicine Blocks Abdominal Aortic Aneurysm Development by Maintaining Vascular Smooth Muscle Cell Homeostasis. Int J Biol Sci. 2024;20(6):2092–110. 38617538 10.7150/ijbs.93544 PMC11008260

[50] Krishna SM, Seto SW, Jose RJ, Li J, Morton SK, Biros E, Wnt Signaling Pathway Inhibitor Sclerostin Inhibits Angiotensin II-Induced Aortic Aneurysm and Atherosclerosis. Arterioscler Thromb Vasc Biol. 2017 Mar;37(3):553–66. 28062506 10.1161/ATVBAHA.116.308723

[51] Schaale K, Brandenburg J, Kispert A, Leitges M, Ehlers S, Reiling N. Wnt6 Is Expressed in Granulomatous Lesions of Mycobacterium tuberculosis–Infected Mice and Is Involved in Macrophage Differentiation and Proliferation. J Immunol. 2013 Nov 1;191(10):5182–95. 24123681 10.4049/jimmunol.1201819

[52] Raffort J, Lareyre F, Clément M, Hassen-Khodja R, Chinetti G, Mallat Z. Monocytes and macrophages in abdominal aortic aneurysm. Nat Rev Cardiol. 2017 Aug;14(8):457–71. 28406184 10.1038/nrcardio.2017.52

[53] Xu J, Liu Z, Yang Q, Ma Q, Zhou Y, Cai Y, Adenosine kinase inhibition protects mice from abdominal aortic aneurysm via epigenetic modulation of VSMC inflammation. Cardiovasc Res. 2024 Sept 2;120(10):1202–17. 38722818 10.1093/cvr/cvae093 PMC11368124

[54] Toghill BJ, Saratzis A, Freeman PJ, Sylvius N, UKAGS collaborators, Bown MJ. SMYD2 promoter DNA methylation is associated with abdominal aortic aneurysm (AAA) and SMYD2 expression in vascular smooth muscle cells. Clin Epigenetics. 2018;10:29. 29507647 10.1186/s13148-018-0460-9 PMC5833080

[55] Skorvanova M, Matakova T, Skerenova M, Sarlinova M, Drobkova H, Petras M, Methylation of MMP2, TIMP2, MMP9 and TIMP1 in abdominal aortic aneurysm. Bratisl Lek Listy. 2020;121(10):717–21. 32955903 10.4149/BLL_2020_117

[56] Kelemen M, Danesh J, Di Angelantonio E, Inouye M, O’Sullivan J, Pennells L, Evaluating the cost-effectiveness of polygenic risk score-stratified screening for abdominal aortic aneurysm. Nat Commun. 2024 Sept 14;15(1):8063. 39277617 10.1038/s41467-024-52452-w PMC11401842

